# Phenotypic Plasticity in Sexual Reproduction Based on Nutrients Supplied From Vegetative Ramets in a *Leymus chinensis* Population

**DOI:** 10.3389/fpls.2019.01681

**Published:** 2020-01-16

**Authors:** Jian Guo, Haiyan Li, Yunfei Yang

**Affiliations:** Key Laboratory of Vegetation Ecology, Ministry of Education, Institute of Grassland Science, Northeast Normal University, Changchun, China

**Keywords:** clonal plant, homogeneous environment, *Leymus chinensis*, phenotypic plasticity, physiological integration, sexual reproduction

## Abstract

Phenotypic plasticity is considered a major mechanism that allows plants to adapt to heterogeneous environments. The physiological integration between the interconnected rhizomes or stolons of clonal plants influences the plasticity of such plants in heterogeneous environments. However, the determinants of plasticity of reproductive ramets in clonal plants in homogeneous environments are unclear. Here, we chose *Leymus chinensis*, a perennial rhizomatous grass, and conducted a series of field experiments *in situ*, including grading sampling of reproductive ramets and different connection forms of vegetative ramets labeled with ^15^N at four reproductive stages. Reproductive ramet biomass, inflorescence biomass, seed number, seed-setting percentage, reproductive allocation, and reallocation significantly increased with an increase in the number of vegetative ramets connected to tillering nodes, and the plasticity indexes of these six phenotypic characteristics showed similar increasing trends. The amount of nutrients supplied from the connected vegetative ramets to the reproductive ramets was significantly affected by the transfer direction, reproductive stage, and position order of the vegetative ramets. Throughout the sexual reproduction stage, nutrients were preferentially transferred to the acropetal reproductive ramet in *L. chinensis* populations. The amount of nutrients supplied from the connected vegetative ramets to the reproductive ramets at the milk-ripe stage, when sexual reproduction was most vigorous, was significantly larger than that at other reproductive stages. The amount of nutrients supplied from the spacer vegetative ramet to the acropetal reproductive ramet was significantly larger than that to the basipetal reproductive ramet. The closer the vegetative ramet was to the reproductive ramet, the more nutrients were supplied; the amount of nutrients supplied was significantly negatively related to the position order of the vegetative ramet. We identified the determinant of plasticity in sexual reproduction in clonal plants in a homogeneous environment: physiological integration between ramets within clones. Our results are vital for better understanding the adaptation of populations and even the evolution of species of clonal plants.

## Introduction

Phenotypic plasticity, or the ability of a given genotype to generate various phenotypes under different environmental conditions (Bradshaw, 1965; Sultan, 2000; Bradshaw, 2006), is considered an important ecological strategy that allows plants to respond quickly to changes in their environment (Nicotra et al., 2010). In the last two decades, the phenotypic plasticity of plants has become a central issue in ecological and evolutionary research. Most studies of phenotypic plasticity have mainly focused on phenotypic responses to abiotic environmental factors, such as light (Wahl et al., 2001; Valladares and Niinemets, 2008), temperature (Atkin et al., 2006; Fraser et al., 2008), nutrients (Wahl et al., 2001), and water (Sultan and Bazzaz, 1993; Nilson and Assmann, 2010). However, potential plasticity to a given abiotic factor can be affected by other biotic factors in complex environments (Stevens and Jones, 2006; Wang et al., 2017a). For example, interspecific competition may limit plant responses to changing environments and thus constrain phenotypic plasticity (Callaway et al., 2003). However, the determinants of the phenotypic plasticity of intraclonal individuals in homogeneous environments still require further research.

Clonal plants are widely distributed in numerous ecosystems, such as grasslands and wetlands (Jónsdóttir and Watson, 1997), and they can produce genetically identical and potentially independent offspring through vegetative propagation to maintain regeneration of the whole population (de Kroon and van Groenendael, 1997). Within a clonal plant, the clonally formed offspring are specifically referred to as “ramets” (Harper, 1977); adjacent ramets are usually interconnected by rhizomes, stolons, or roots, and these physical connections enable plants to translocate and share resources such as water, mineral nutrients, and photoassimilates, a phenomenon also known as physiological integration (Ashmun et al., 1982; Alpert, 1996). Many studies have shown that physiological integration can enhance or reduce plasticity or can even induce novel plastic responses (de Kroon and Hutchings, 1995; Stuefer et al., 1996; Alpert, 1999b; de Kroon et al., 2005; Xu et al., 2010; Xu et al., 2012; Roiloa and Hutchings, 2013), but these studies have concentrated only on morphology, growth, and physiology. Sexual reproduction is an essential part of the life history of all plants (Richards, 1986). Sexual reproduction may be greatly beneficial in heterogeneous or changing environments due to the production of genetically diverse offspring (e.g., Maynard Smith, 1971). Furthermore, sexual reproduction also helps offspring spread over long distances, thereby expanding the population constantly (Eckert and Barrett, 2002). Therefore, plasticity in sexual reproduction plays a crucial role in the adaptation and evolution of clonal plants. However, how physiological integration affects plasticity in sexual reproduction of clonal plants has not been reported thus far.

Previous studies concerning the effect of physiological integration on the plasticity of clonal plants were mostly carried out in heterogeneous environments. Moreover, what has been confirmed relies on the heterogeneity of the environment (Alpert, 1999b). Current studies on physiological integration in heterogeneous environments generally suggest that integration has no effect on clonal performance when resource availability is spatially uniform (e.g., Evans and Whitney, 1992; Alpert, 1999a). In fact, clonal fragments often contain ramets that are at different developmental stages or belong to different types, such as parental ramets and daughter ramets or vegetative ramets and reproductive ramets. For example, some studies found that the benefits of physiological integration in a homogeneous environment existed both between ramets of different ages with developmental differences (Stuefer, 1998; Yu et al., 2002; Dong et al., 2015; Zhang et al., 2016) and between ramets of different types with production differences (Wang et al., 2017b). Therefore, even under the same levels of external resource supply, resource sharing between interconnected ramets might improve clonal performance owing to differences in the amounts of absorbed or assimilated resources. Intraspecific phenotypic variation in plants has been extensively studied in homogeneous environments (e.g., Rutter and Fenster, 2007; Bell and Galloway, 2008; Leiblein-Wild and Tackenberg, 2014). However, it is unclear what role the physiological integration among intraclonal ramets may play in plasticity in sexual reproduction in homogeneous environments.

*Leymus chinensis*, a typical clonal plant with vigorous belowground rhizomes (Yang et al., 1995; Wang and Ba, 2008), is widely distributed in eastern areas of the Eurasian steppe. In natural grasslands, *L. chinensis* mainly relies on vegetative propagation for spatial expansion and population renewal (Yang et al., 1995), and the seed-setting percentage from sexual reproduction is approximately 25% (Duan and Fan, 1984). The phenotypic characteristics of sexual reproduction in *L. chinensis* vary greatly under natural conditions. The minimum seed number per inflorescence is 0, and the maximum is 45, whereas the minimum seed-setting percentage is 0, and the maximum is 67.5% (Li et al., 2004). Until now, studies concerning physiological integration in *L. chinensis* have been restricted to the connection between ramets enhancing the plastic response of photosynthetic physiology or biomass to different levels of nutrients or resources (Gao et al., 2014; Zhou et al., 2014). However, a comprehensive understanding of the effects of different connection forms and differences in the number of connected vegetative ramets on the plasticity of reproductive ramets in *L. chinensis* clones is lacking.

In this study, we chose *L. chinensis* and conducted a series of field and laboratory experiments including grading sampling of reproductive ramets and different connection forms of vegetative ramets labeled with ^15^N at four reproductive stages. The objectives of our study were as follows: a) to verify that the plasticity in sexual reproduction in *L. chinensis* clones can be induced by differences in nutrient supply from the connected vegetative ramets in a natural homogeneous environment and b) to reveal the patterns of nutrient supply from the connected vegetative ramets to reproductive ramets in *L. chinensis* clones. We hypothesize that 1) the phenotypic characteristics and plasticity in these characteristics of reproductive ramets will increase with an increase in the number of vegetative ramets connected to tillering nodes; 2) the amount of nutrients supplied from vegetative ramets with various connection forms to the reproductive ramets will be largest at the vigorous milk-ripe stage; 3) the amount of nutrients supplied from spacer vegetative ramets to acropetal reproductive ramets will be much larger than that to basipetal reproductive ramets; and 4) the closer the vegetative ramet is to the reproductive ramet, the more nutrients will be translocated.

## Materials and Methods

### Study Area

The study was conducted at the Grassland Ecological Research Station of Northeast Normal University in the southern Songnen Plain in Changling County, Jilin Province, Northeast China (44°38′N, 123°41′E), in 2018, the fourth year after the experimental plots were set up. This area is characterized by a semi-humid, semi-arid, and temperate continental monsoonal climate with hot and rainy summers and cold and dry winters. The annual mean temperature varies from 4.6 to 6.4°C. The number of days with an average temperature ≥10°C ranges from 120 to 140. The annual accumulated temperature varies from 3,000 to 3,500°C. The annual mean precipitation ranges from 300 to 450 mm, with the majority concentrated in June-September, and the annual evaporation varies from 1,200 to 1,400 mm. The frost-free period is approximately 130–165 days (Li et al., 2018).

### Study Species

*L. chinensis* (Trin.) Tzvel., a perennial rhizomatous C_3_ grass, is widely distributed in the eastern Eurasian steppe, including northwestern Siberia, western North Korea, the People’s Republic of Mongolia, the Inner Mongolian Plateau, and the Northeast Plain of China (Kuo, 1987). As the major dominant species in the grasslands of Northeast China, *L. chinensis* is often used as a forage grass because of its high nutritional value and good palatability (Zhu, 2004). *L. chinensis* has strong adaptability to unfavorable environmental conditions, including salt, alkalinity, drought, and low temperature (Jin et al., 2008; Chen et al., 2013; Zhai et al., 2014), and this grass shows extensive plasticity in morphological and physiological characteristics. In natural grasslands, *L. chinensis* mainly relies on the vegetative propagation of rhizomes for population expansion (Yang et al., 1995), and its capacity for sexual reproduction is rather weak. The ramets of *L. chinensis* are interconnected by rhizomes or tillering nodes (which refer to unelongated basal internode of ramets). Most often, only one of the ramets interconnected by tillering nodes undergoes sexual reproduction, flowering and seed set, called reproductive ramets; the others only conduct vegetative growth and do not produce flowers, called vegetative ramets. In the Songnen grassland, *L. chinensis* usually begins turning green in early April, heads in the middle or later period of May, flowers in June, and ripens seeds in mid-July (Zhu, 2004).

### Experimental Platform

In May of 2015, a total of 30 experimental plots were established. Each plot had an area of 4 m^2^ (2 m × 2 m), and adjacent plots were at least 2 m apart. Similarly sized vegetative ramets of *L. chinensis* (height: 20.9 ± 0.3 cm, mean ± SE) were collected from a natural clone in the study area, and a group of nine ramets was transplanted into each plot with rows 0.5 m apart and ramets 0.5 m apart. All the experimental plots were manually irrigated for several days after transplanting to ensure that the ramets survived. No fertilizer was added to the soil. *L. chinensis* ramets were not obviously affected by diseases or insect pests throughout the study, and all other plants were regularly pulled by hand from 2015 to 2018. The soil type was sandy loam. The soil of the top 20-cm-thick layer was homogenous, with a total N of 1.01 g kg^−1^, an organic C of 6.23 g kg^−1^, a total P of 0.74 g kg^−1^, a pH of 8.37, and an electrical conductivity of 70.85 μS cm^−1^.

### Effects of the Number of Vegetative Ramets Connected to Tillering Nodes on Phenotypic Characteristics of Reproductive Ramets

To investigate the effects of the number of vegetative ramets connected to tillering nodes on the phenotypic characteristics of reproductive ramets in a natural homogeneous environment, a marking manipulation was conducted at the early heading stage of *L. chinensis* in 2018. Reproductive ramets whose inflorescence top reached approximately 2 cm over the flag leaf sheath and that were interconnected with 1, 2, 3, and ≥4 vegetative ramets by tillering nodes were marked with tags. For each number in the gradient, in each plot, only one reproductive ramet was marked, for a total of 25 reproductive ramets in 25 plots. Five plots were disqualified because the desired number gradient was unavailable. All marked reproductive ramets were harvested together at the dough stage. Reproductive ramet height, floret number, and seed number were measured. Each reproductive ramet was separated into leaves, stem, inflorescence, and seeds. Dry biomass, including all parts of a reproductive ramet, was determined after oven-drying to a constant weight at 65°C.

### Effects of Transfer Direction, Reproductive Stage, and Position Order on Nutrient Translocation

**Figure 1:**
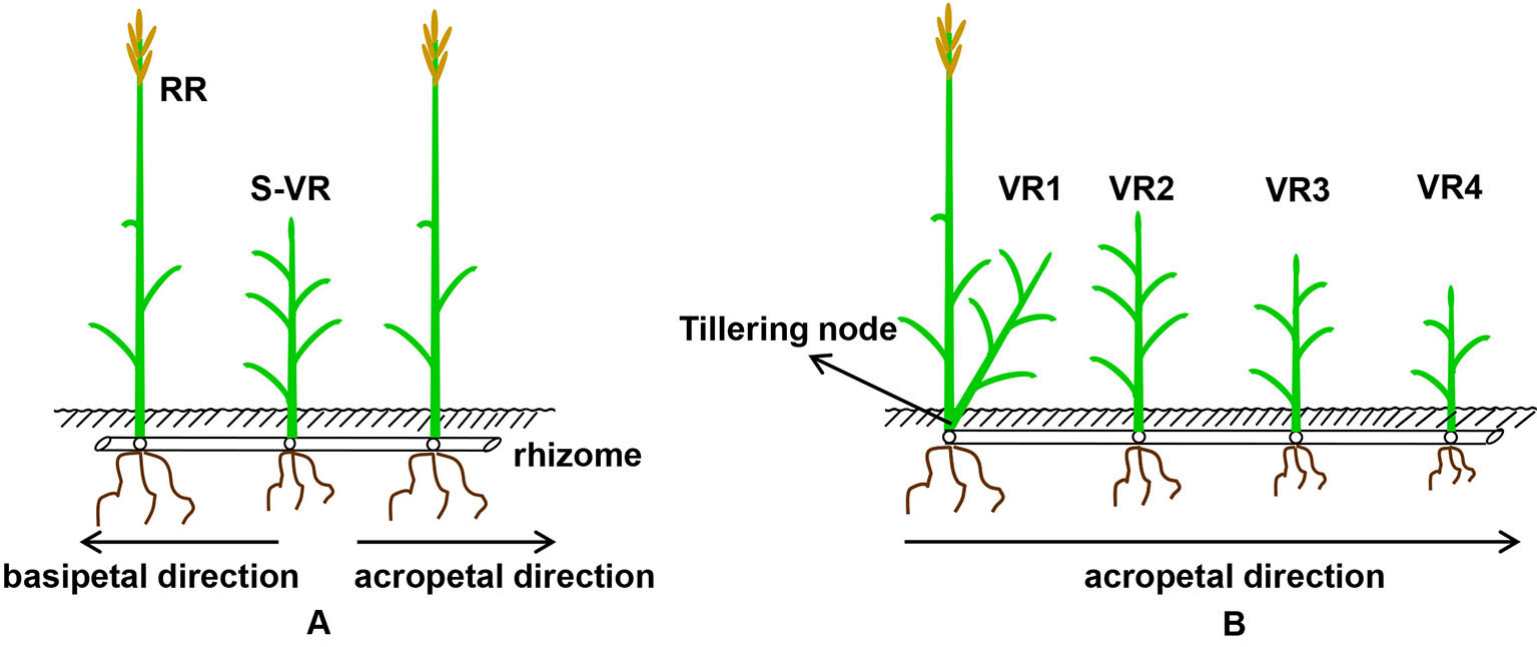
A diagrammatic drawing of spacer vegetative ramets (**A**, three-member-clonal system of two reproductive ramets and one spacer vegetative ramet connected by one and the same rhizome) and acropetal vegetative ramets of different position order (**B**, five-member-clonal system of one basipetal reproductive ramet and four vegetative ramets connected *via* one and the same rhizome) labeled with ^15^N isotope (RR, reproductive ramet; S, spacer; VR, vegetative ramets; digital 1, 2, 3, 4, position order of acropetal vegetative ramet, VR1 is on the tillering node, VR2, VR3, and VR4 are all on the rhizome).

**Figure 2:**
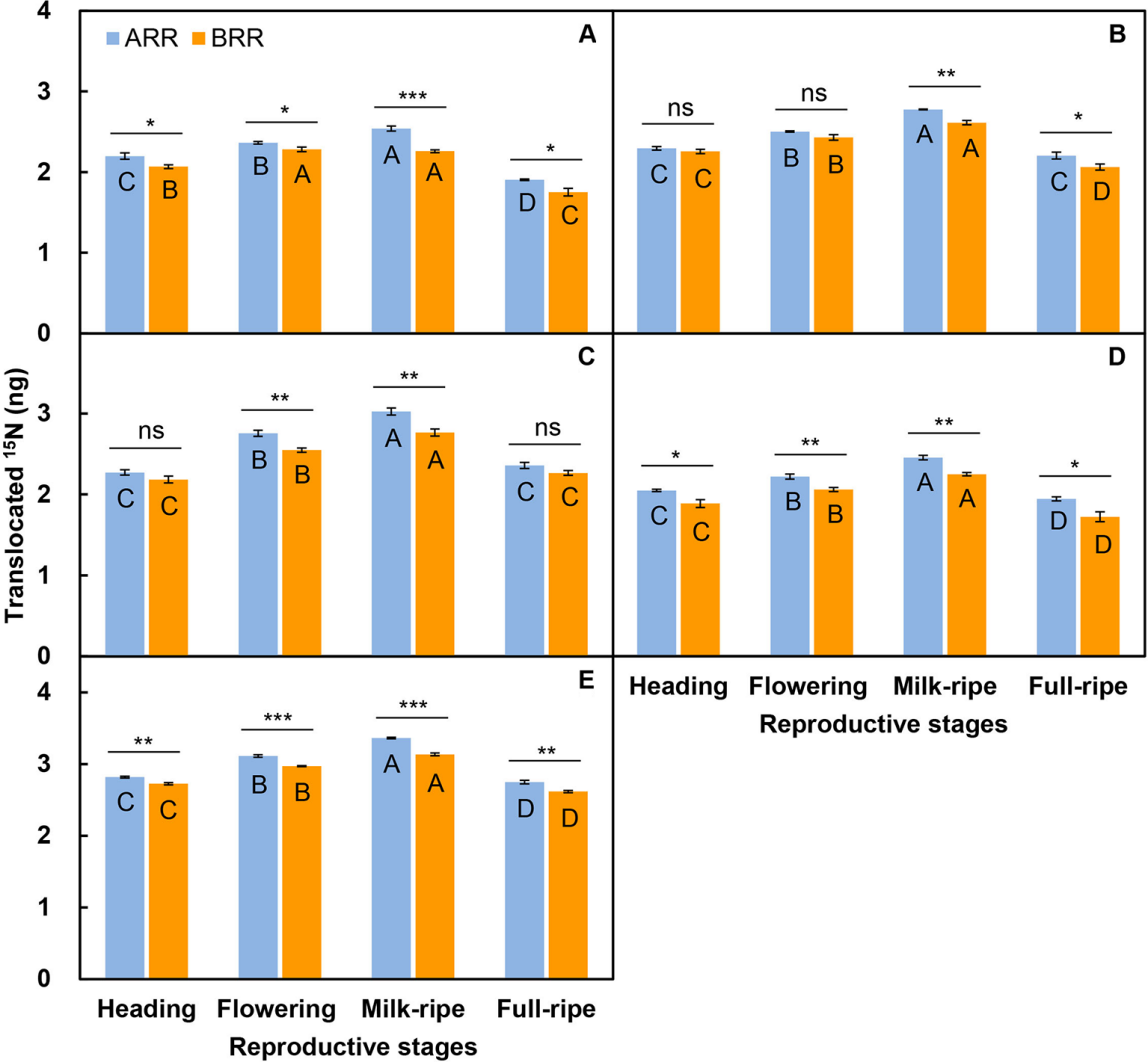
Comparison on the amounts of translocated ^15^N from labeled vegetative ramets toward the leaf blade **(A)**, sheath **(B)**, stem **(C)**, inflorescence **(D)**, and whole ramet (**E**) of reproductive ramets both between two directions (ARR, acropetal reproductive ramet; BRR, basipetal reproductive ramet) and among four reproductive stages. Symbols for the same reproductive stage indicate significant differences between two directions: ns, *P* > 0.05; *, 0.01< *P* < 0.05; **, 0.001< *P* < 0.01; ***, *P* < 0.001; different *capital letters* for the same directional reproductive ramet indicate significant differences (*P* < 0.05) among reproductive stages. Values are means ± standard error (SE). Note that the *y*-axes are log scales.

To estimate the effects of transfer direction (acropetal and basipetal), reproductive stage (heading, flowering, milk-ripe, and full-ripe stages, the specific definition standard was shown in **Table 1**), and position order of the connected vegetative ramets on the amount of translocated nutrients, a manipulative *in situ*-labeling experiment (according to Putz et al., 2011) was conducted in 2018. At each reproductive stage, five labeling modes were set up: 1) the spacer vegetative ramet located between the two reproductive ramets was labeled with ^15^N (**Figure 1A**); 2) the first, second, third, and fourth acropetal vegetative ramets (the first located at the tillering node of a reproductive ramet, while the others located on the rhizome) were labeled with ^15^N, respectively (**Figure 1B**). The labeling experiment at each reproductive stage followed a randomized block design, and each plot was treated as a block. Because *L. chinensis* relied on the vegetative propagation of rhizomes to expand toward the edge of the plots, the aboveground ramets developing from rhizome node buds arranged themselves in a linear pattern. Within each plot, all the labeling modes were randomly assigned to the edge of the plot. Adjacent labeled ramets were at least 50 cm apart to avoid cross-contamination. At each reproductive stage, four plots were randomly selected for ^15^N labeling, and the rest of the labeling was conducted in four plots randomly selected from the remaining nonlabeled plots.

**Table 1:** Specific definition standard of four reproductive stages in *Leymus chinensis*.

Reproductive stage	Specific definition standard
Heading stage	The base of the inflorescence was completely exposed from the flag leaf sheath.
Flowering stage	Both the palea and lemma of the florets on the whole inflorescence dehisced, and the anthers spread pollen.
Milk-ripe stage	The seeds started to deposit large amounts of nutrient and seed inclusions appeared as a white paste.
Full-ripe stage	The seeds hardened to appear yellow.

Each ^15^N labeling experiment was conducted during a succession of sunny days. Prior to labeling, the soil surface was covered with plastic wrap to avoid soil contamination. For each vegetative ramet, 1 ml of ^15^N-labeled urea solution (made at the Shanghai Research Institute of Chemical Industry, Shanghai, China) was applied, with a urea concentration of 2% g ml^−1^ and a ^15^N abundance of 5.18%. The urea solution was daubed gently with a small paintbrush from the base to the tip of the leaf surface to ensure that the total solution was smeared evenly on both sides of the leaf. During brushing, leaves were held with forceps. Labeling was applied once per day over three consecutive days, and meticulous operation with extra care was required to prevent the urea solution from contacting any part of the reproductive ramet or soil. For the control treatment, the ^15^N-labeled urea solution was replaced by an equal volume of distilled water.

At each reproductive stage, the reproductive ramets that were connected to the labeled vegetative ramets were harvested 2 days after labeling. Each ramet was separated into leaf blades, sheath, stem, and inflorescence. The enzyme activity of each part was halted at 105°C for 30 min, and the tissue was dried to a constant weight at 65°C and ground to a fine powder using a ball mill (MM 400 Retsch, Haan, Germany). Approximately 3 mg of sample powder was loaded into a capsule and analyzed using an Elementar Vario EL Cube (Elementar, Langenselbold, Germany) coupled to an isotope ratio mass spectrometer (Isoprime 100, Elementar, Langenselbold, Germany), with an overall precision greater than 0.2‰. The isotope value was used the standard delta (*δ*) notion and expressed as follows:

(1) $$\delta^{15}\text{N (}‰\text{) }=\;\left(R_{\text{sample}}/R_{\text{standard}}-\;1\right)\;\times \;1,000$$

where R_sample_ is the ^15^N/^14^N in a sample and R_standard_ is the ^15^N/^14^N in atmospheric N_2_. As a universal standard for ^15^N, we used atmospheric air (*R*_standard_ = 0.00368).

To calculate the amount of ^15^N from labeling, the *F*-ratio was calculated as follows:

(2) $$F-\text{ratio }=\;\left[R_{\text{sample}}/\left(R_{\text{sample}}+\;1\right)\right]\;\times \;100$$

The amount of translocated ^15^N in each sample was then calculated as follows:

(3) $$\text{translocated}{\;}^{15}\text{N }=W_{\text{dry}}\times B\times \;\left(F_{\text{S}}-F_{\text{U}}\right)$$

where *W*_dry_ is the dry weight of a sample, *B* is the nitrogen content per unit mass of a sample, *F*_S_ is the *F*-ratio of a sample, and *F*_U_ is the mean *F*-ratio of four unlabeled control ramets for each harvest.

### Data Analysis

All statistical analyses were performed using SPSS 22.0 software (SPSS Inc., Chicago, IL, USA). All data were tested for a normal distribution and homogeneous variances through a Kolmogorov-Smirnov test and Levene’s test, respectively. The significance level was set at *P* < 0.05.

One-way ANOVA was conducted to assess the effect of the number of connected vegetative ramets on the phenotypic characteristics of reproductive ramets. The seed-setting percentage was calculated as the percentage of seeds relative to the number of florets, reproductive allocation was calculated as the percentage of inflorescence biomass relative to ramet biomass, and reproductive reallocation was calculated as the percentage of seed biomass relative to ramet biomass. To evaluate the phenotypic plasticity in each characteristic of reproductive ramets interconnected with different number grades of vegetative ramets by tillering nodes, the phenotypic plasticity index (PPI) was calculated as (Valladares et al., 2000; Valladares et al., 2006) follows:

(4) PPI=(maxT−minT)/maxT

where *T* is the characteristic mean value in each number gradient.

Two-way ANOVA was performed to assess the effects of transfer direction and reproductive stage on the amount of translocated ^15^N in the leaf blade, sheath, stem, inflorescence, and whole ramet of the reproductive ramet and to investigate the effects of position order and reproductive stage on the amount of translocated ^15^N in the leaf blade, sheath, stem, inflorescence, and whole ramet of the reproductive ramet. All data on the amount of translocated ^15^N were log-transformed to meet model assumptions. An independent samples *t*-test and Duncan’s multiple-range test were used to test for significant differences between means of two and more than two groups, respectively. Estimation of regression curves was used to quantify the relationships between the amount of translocated ^15^N in the leaf blade, sheath, stem, inflorescence, and whole ramet of the reproductive ramet and position order of the acropetal vegetative ramet at the heading stage, flowering stage, milk-ripe stage and full-ripe stage with linear, exponential, power, and logarithmic functions. The model with the lowest Akaike information criterion (AIC) value was selected as the best (Sugiura, 1978; Hurvich and Tsai, 1991).

## Results

### Effects of the Number of Vegetative Ramets Connected to Tillering Nodes on Phenotypic Characteristics of Reproductive Ramets

Ramet biomass, inflorescence biomass, seed number, seed-setting percentage, reproductive allocation, and reallocation as well as the PPIs of these traits in *L. chinensis* tended to increase with an increase in the number of vegetative ramets connected to tillering nodes, while ramet height and its PPI showed basically no change (**Table 2**). Overall, the PPI of ramet height was the smallest, while that of seed number per inflorescence was the largest (**Table 2**). The biomass of reproductive ramets connected to ≥ four vegetative ramets was significantly larger than that of ones connected to one or two vegetative ramets. The inflorescence biomass, seed number, reproductive allocation, and reallocation of reproductive ramets connected to at least three vegetative ramets were significantly greater than those of ones connected to one or two vegetative ramets. The seed-setting percentage of reproductive ramets connected to at least two vegetative ramets was significantly larger than that of ones connected to one vegetative ramet (**Table 2**). These results indicated that the number of vegetative ramets connected to tillering nodes had a significant effect on the phenotypic characteristics and plasticity in such characteristics (except for ramet height) of reproductive ramets.

**Table 2:** Phenotypic characteristics of the reproductive ramets connected with different number grades of vegetative ramets by tillering nodes in the *Leymus chinensis* population (sample size, *n*).

Characteristics	Grade	*n*	Max.	Min.	Mean	SD	PPI
Height (cm)	1	25	88.0	49.6	70.3 a	10.0	0.44
	2	25	81.5	52.1	68.9 a	8.0	0.36
	3	25	88.6	57.4	69.8 a	9.0	0.35
	≥4	25	99.2	53.6	68.5 a	9.9	0.46
	Total	100	99.2	49.6	69.4	9.2	0.50
Total biomass (g)	1	25	1.036	0.392	0.659 b	0.196	0.62
	2	25	1.099	0.277	0.629 b	0.186	0.75
	3	25	1.240	0.465	0.735 ab	0.188	0.63
	≥4	25	1.775	0.456	0.803 a	0.291	0.74
	Total	100	1.775	0.277	0.707	0.227	0.84
Inflorescence biomass (g)	1	25	0.277	0.105	0.173 b	0.043	0.62
	2	25	0.300	0.070	0.169 b	0.049	0.77
	3	25	0.403	0.140	0.223 a	0.074	0.65
	≥4	25	0.590	0.108	0.252 a	0.119	0.82
	Total	100	0.590	0.070	0.204	0.084	0.88
Seed number (seeds)	1	25	44	14	27.3 b	7.3	0.68
	2	25	54	15	29.8 b	9.4	0.72
	3	25	64	21	44.1 a	11.3	0.67
	≥4	25	146	19	58.2 a	31.1	0.87
	Total	100	146	14	39.8	21.3	0.90
Seed-setting percentage	1	25	30.4	16.5	23.3 c	4.5	0.46
	2	25	37.8	17.6	28.5 b	5.9	0.53
	3	25	56.4	20.8	36.0 a	8.5	0.63
	≥4	25	69.4	24.7	42.2 a	13.9	0.64
	Total	100	69.4	16.5	32.5	11.4	0.76
Reproductive allocation (%)	1	25	36.5	18.6	26.9 c	4.4	0.49
	2	25	38.5	16.0	27.3 bc	5.5	0.58
	3	25	47.3	18.8	30.5 ab	6.8	0.60
	≥4	25	48.0	14.9	31.0 a	7.0	0.69
	Total	100	48.0	14.9	28.9	6.2	0.69
Reproductive reallocation (%)	1	25	46.7	22.0	33.9 b	7.6	0.53
	2	25	55.4	27.3	38.4 b	6.4	0.51
	3	25	63.7	32.4	45.9 a	8.0	0.49
	≥4	25	77.3	29.9	52.3 a	14.4	0.61
	Total	100	77.3	22.0	42.6	11.8	0.72

Different *small letters* for the same trait indicate significant differences (*P* < 0.05) among grades.

### Effects of Transfer Direction and Reproductive Stage on Nutrient Translocation

For the three-member-clonal system of two reproductive ramets and one spacer vegetative ramet connected by one and the same rhizome, the amount of translocated ^15^N in the leaf blade, sheath, stem, inflorescence, and whole ramet was significantly affected by the transfer direction and reproductive stage. The effects of transfer direction on the amount of translocated ^15^N in the leaf blade and whole ramet changed significantly with reproductive stage (**Table 3**). The amount of translocated ^15^N in all parts of the acropetal reproductive ramet was much larger than that in the corresponding parts of the basipetal ramet. The amount of translocated ^15^N in the leaf blade, inflorescence and whole ramet of the acropetal reproductive ramet was significantly larger than that in the corresponding parts of the basipetal ramet at each reproductive stage (**Figures 2A, D, E**); however, this pattern appeared at the milk-ripe and full-ripe stages for sheath and occurred at the flowering and milk-ripe stages for stem (**Figures 2B, C**). These results indicated that nutrients were preferentially transferred to the acropetal reproductive ramet in *L. chinensis* populations during sexual reproduction. With the progression of sexual reproduction, the amount of translocated ^15^N in all parts of both the acropetal and basipetal reproductive ramets first increased and then decreased, and the amount in all parts was significantly larger at the milk-ripe stage than at the other three stages (**Figure 2**), indicating that the amount of translocated nutrients was largest at the milk-ripe stage, when sexual reproduction was most vigorous, in the *L. chinensis* population.

**Table 3:** Two-way ANOVA for the effects of transfer direction, reproductive stage, and their interaction on amounts of ^15^N (log-transformed) translocated from labeled vegetative ramets toward the leaf blade, sheath, stem, inflorescence, and whole ramet of reproductive ramets.

Variable	Transfer direction (TD)		Reproductive stage (RS)		TD × RS	
	*F*_1,24_	*P*	*F*_3,24_	*P*	*F*_3,24_	*P*
Leaf blade ^15^N	64.43	<0.001	159.43	<0.001	4.50	0.012
Sheath ^15^N	27.98	<0.001	151.00	<0.001	2.22	0.112
Stem ^15^N	38.58	<0.001	141.22	<0.001	2.69	0.069
Inflorescence ^15^N	58.35	<0.001	83.24	<0.001	0.41	0.750
Whole ramet ^15^N	164.65	<0.001	498.32	<0.001	6.24	0.003

### Effects of Position Order and Reproductive Stage on Nutrient Translocation

**Figure 3:**
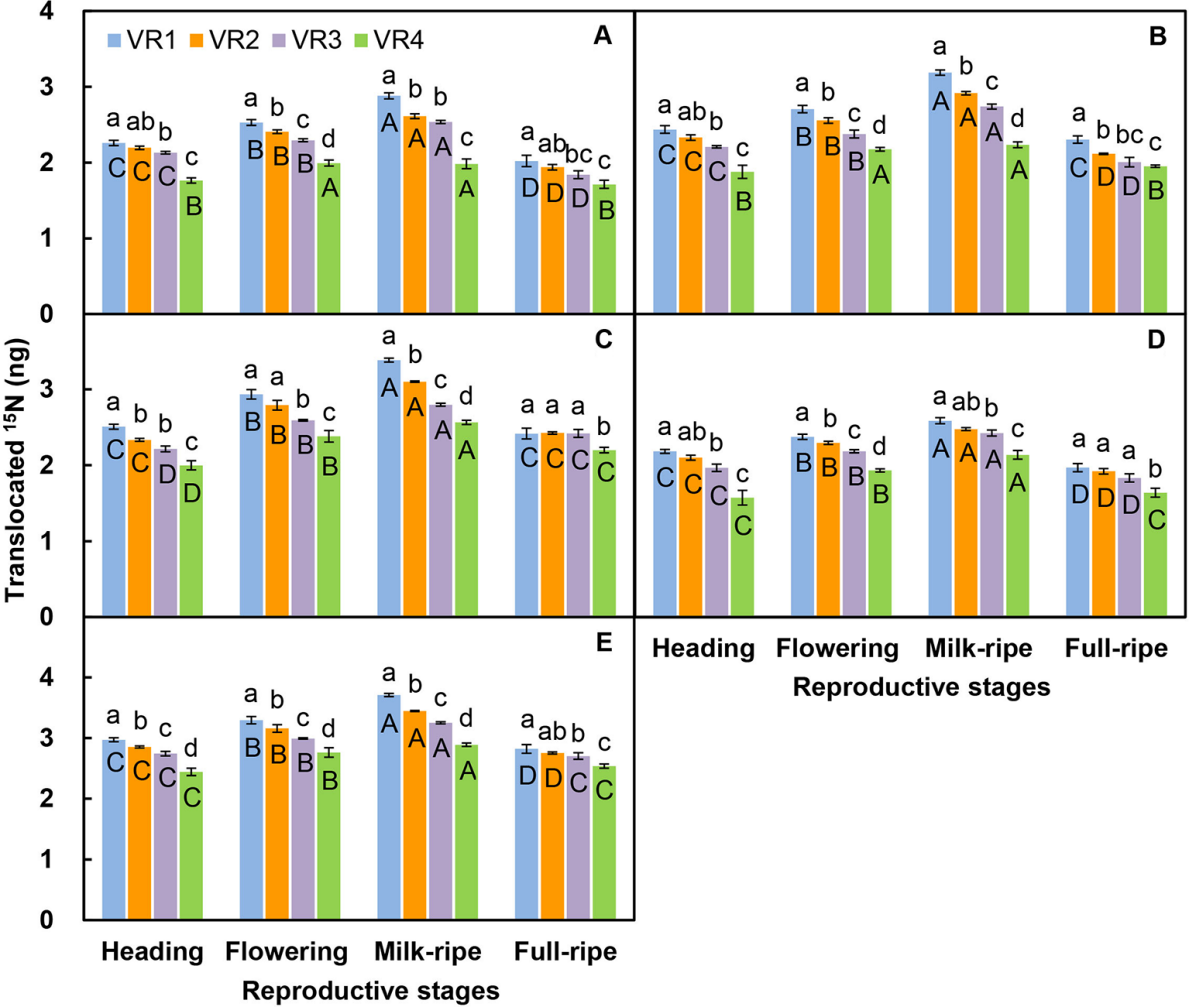
Comparison on the amounts of translocated ^15^N from labeled vegetative ramets toward leaf blade **(A)**, sheath **(B)**, stem **(C)**, inflorescence **(D)**, and whole ramet **(E)** of basipetal reproductive ramets among the four treatments of position order (VR1, the first acropetal vegetative ramet located on the tillering node; VR2, VR3, VR4, the second, third, and fourth acropetal vegetative ramets located on the rhizome) and among the four reproductive stages. Different *small letters* for the same stage indicate significant differences (*P* < 0.05) among position orders, and different *capital letters* for the same position order indicate significant differences (*P* < 0.05) among reproductive stages. Values are means ± standard error (SE). Note that the *y*-axes are log scales.

**Figure 4:**
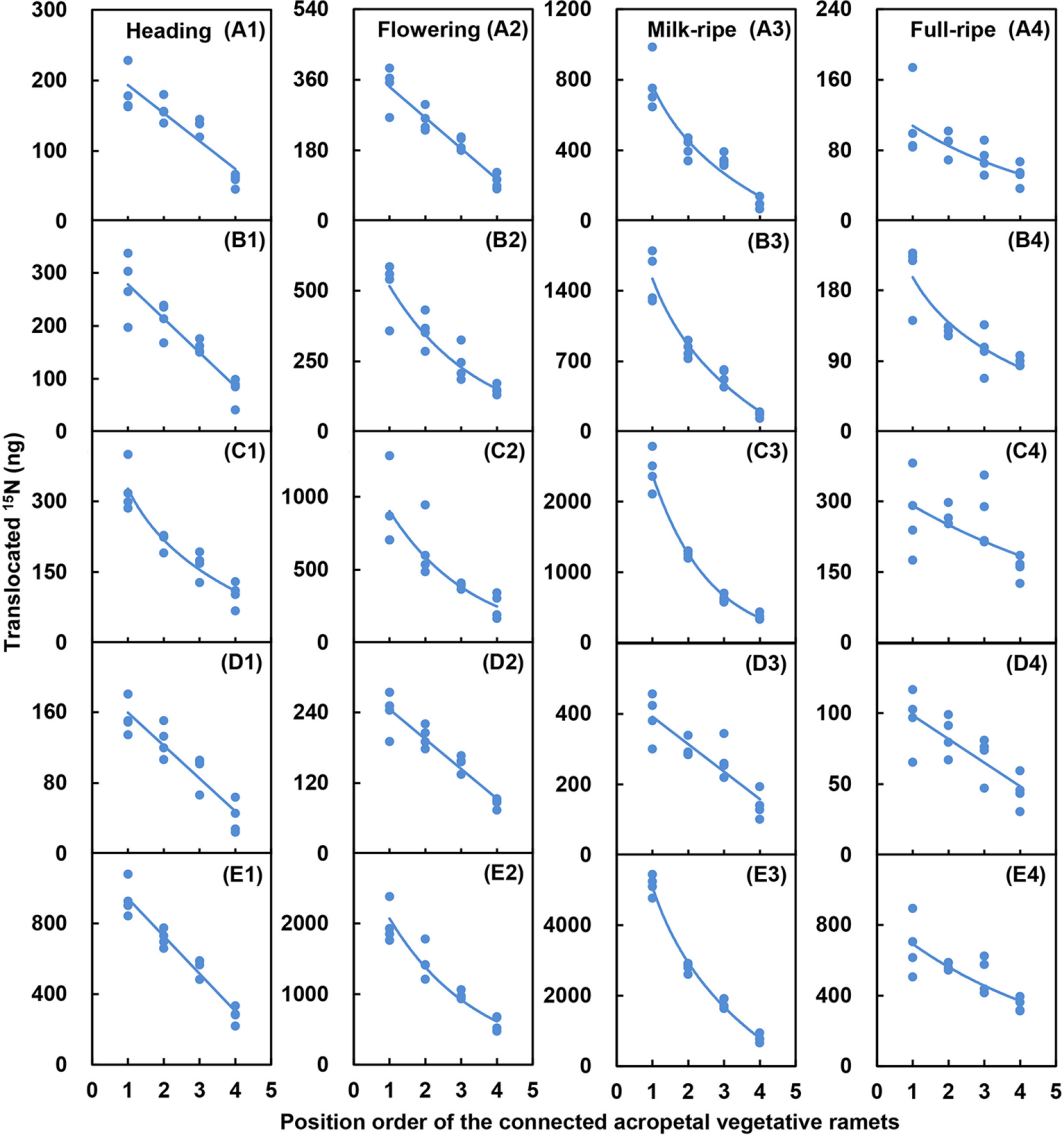
Relationships between the amounts of translocated ^15^N in the leaf blade (A1–A4), sheath (B1–B4), stem (C1–C4), inflorescence (D1–D4), and whole ramet (E1–E4) of reproductive ramets and the position order of the connected acropetal vegetative ramets at heading stage, flowering stage, milk-ripe stage and full-ripe stage, respectively. The regression lines are based upon regression analysis.

For the five-member-clonal system of one basipetal reproductive ramet and four vegetative ramets connected *via* one and the same rhizome, the amount of translocated ^15^N in the leaf blade, sheath, stem, inflorescence, and whole ramet of the basipetal reproductive ramet was significantly affected by the position order of the vegetative ramet and reproductive stage. The position order and reproductive stage interacted significantly to affect the amount of translocated ^15^N in the leaf blade, sheath, stem, and whole ramet (**Table 4**). For each reproductive stage, with increasing position order of the connected acropetal vegetative ramet, the amount of translocated ^15^N in all parts of the basipetal reproductive ramet significantly decreased by a linear, exponential, or logarithmic function (**Figures 3** and **4**, **Table 5**), indicating that the closer the vegetative ramet was to the reproductive ramet, the more nutrients were translocated. With the progression of sexual reproduction, the amount of translocated ^15^N in all parts of the basipetal reproductive ramet first increased and then decreased, and the amount in all parts was significantly larger at the milk-ripe stage than at the other three stages (**Figure 3**).

**Table 4:** Two-way ANOVA for the effects of position order, reproductive stage and their interaction on amounts of ^15^N (log-transformed) translocated from labeled vegetative ramets toward the leaf blade, sheath, stem, inflorescence, and whole ramet of basipetal reproductive ramets.

Variable	Position order (PO)		Reproductive stage (RS)		PO × RS	
	*F*_3,48_	*P*	*F*_3,48_	*P*	*F*_9,48_	*P*
Leaf blade ^15^N	135.69	<0.001	174.09	<0.001	6.77	<0.001
Sheath ^15^N	137.76	<0.001	190.60	<0.001	7.22	<0.001
Stem ^15^N	102.97	<0.001	204.07	<0.001	7.09	<0.001
Inflorescence ^15^N	76.00	<0.001	121.49	<0.001	1.37	0.231
Whole ramet ^15^N	256.11	<0.001	403.12	<0.001	10.65	<0.001

**Table 5:** Regression equations among translocated ^15^N (ng) in different parts of basipetal reproductive ramets and the position order of the connected acropetal vegetative ramets at four reproductive stages (*n* = 16).

Variable	Reproductive stage	Equation	*R* ^2^	*P*
Leaf blade^15^N	Heading	y = 233.098–39.688x	0.806	<0.001
	Flowering	y = 420.958–78.678x	0.895	<0.001
	Milk-ripe	y = 766.702–452.696lnx	0.882	<0.001
	Full-ripe	y = 136.571e^−0.236x^	0.582	0.001
Sheath ^15^N	Heading	y = 342.545–64.322x	0.830	<0.001
	Flowering	y = 774.968e^−0.407x^	0.875	<0.001
	Milk-ripe	y = 1518.871–949.333lnx	0.937	<0.001
	Full-ripe	y = 196.670–82.762lnx	0.783	<0.001
Stem ^15^N	Heading	y = 326.697–156.233lnx	0.885	<0.001
	Flowering	y = 1383.485e^−0.429x^	0.803	<0.001
	Milk-ripe	y = 4489.455e^−0.636x^	0.983	<0.001
	Full-ripe	y = 338.471e^−0.151x^	0.333	0.019
Inflorescence ^15^N	Heading	y = 196.656–37.152x	0.846	<0.001
	Flowering	y = 295.759–50.587x	0.891	<0.001
	Milk-ripe	y = 469.413–77.725x	0.776	<0.001
	Full-ripe	y = 115.009–16.627x	0.628	<0.001
Whole ramet ^15^N	Heading	y = 1154.694–213.094x	0.937	<0.001
	Flowering	y = 3104.459e^−0.405x^	0.920	<0.001
	Milk-ripe	y = 5075.126–3088.740lnx	0.987	<0.001
	Full-ripe	y = 853.350e^−0.209x^	0.683	<0.001

## Discussion

### Phenotypic Plasticity in Sexual Reproduction in Clonal Plants

Phenotypic plasticity is usually triggered by environmental cues (Bradshaw, 1965;  Bradshaw, 2006), and it allows plant individuals or populations to make optimal adjustments to environmental heterogeneity (Richter et al., 2012). In clonal plants, physiological integration can modify or induce the plasticity of ramets in heterogeneous environments, but it is unclear whether physiological integration plays the same role in sexual reproduction in homogeneous environments. In the present study, we found that ramet biomass, inflorescence biomass, seed number, seed-setting percentage, reproductive allocation, and reallocation increased with an increase in the number of vegetative ramets connected to tillering nodes (**Table 2**), which supported our first hypothesis. In our experiment, we first ensured that all ramets of *L. chinensis* originally collected from one clone in each plot were genetically identical. Second, the clones grew in a homogeneous environment, and there were no extreme weather events, pests, or diseases throughout the experiment. Finally, we chose reproductive ramets with synchronous heading as experimental samples, thereby excluding the effect of asynchronous heading on sexual reproduction (Li et al., 2018). Therefore, we were able to confirm that the phenotypic plasticity of reproductive ramets was induced directly by the difference in the number of vegetative ramets connected to tillering nodes. When the connected vegetative ramets were labeled with ^15^N, a certain amount of translocated ^15^N was detected in the reproductive ramets (**Figures 2** and **3**), which further confirmed that vegetative ramets indeed supply nutrients to reproductive ramets. A difference in the number of connected vegetative ramets affected the nutrient supply. The greater the number of vegetative ramets connected to the reproductive ramet was, the greater the nutrient supply was and the better performance of the reproductive ramet was, confirming that physiological integration within a clonal system in a homogeneous environment regulates phenotypic plasticity in sexual reproduction. In addition to well-known external environmental factors, intraclonal integrated regulation also plays an important role in phenotypic plasticity, refreshing our understanding of cues that trigger phenotypic differences.

The PPI is an important indicator used to quantify plasticity in phenotypic characteristics (Valladares et al., 2000; Valladares et al., 2006). Low PPIs indicate low amounts of plasticity, and high PPIs indicate high amounts of plasticity. We found that the PPIs of ramet biomass, inflorescence biomass, seed number, seed-setting percentage, reproductive allocation, and reallocation increased with an increase in the number of vegetative ramets connected to tillering nodes (**Table 2**), which supported our first hypothesis. Under natural conditions, a size hierarchy caused directly or indirectly by variation in growth rates due to factors such as age differences, genetic variation, or environmental heterogeneity exists in most plant populations (Weiner and Solbrig, 1984). In *L. chinensis* populations in a homogeneous environment, an inconsistency in emergence timing causes a size hierarchy of vegetative ramets connected to tillering nodes. Therefore, the greater the number of vegetative ramets connected to the reproductive ramets was, the larger the differences between vegetative ramets in samples of the same grade were and the larger the differences in nutrients supplied to the reproductive ramets were. The plasticity in all phenotypic characteristics of reproductive ramets connected with ≥4 connected vegetative ramets was the greatest (**Table 2**). Furthermore, the growth time of each phenotypic characteristic of reproductive ramets was different. Under natural conditions, the reproductive ramets of *L. chinensis* stopped growing vertically before flowering in mid-June, while seed biomass increased until the full-ripe stage in mid-July, indicating that the growth time of seeds was longer than that of ramet height. Differences in the external environment and nutrient supply may lead to greater plasticity in phenotypic characteristics related to seeds than in ramet height.

Previous studies on the effects of physiological integration mostly involved pot experiments in greenhouses and used two major approaches: the severing approach and the homogeneous-heterogeneous approach (Stuefer et al., 1994; Jónsdóttir and Watson, 1997). The severing of the physical connection between ramets may cause physiological stress and make ramets more susceptible to pathogen infections; thus, the severing approach may overestimate the effects of physiological integration (Jónsdóttir and Watson, 1997). Because resource translocation between ramets also exists under homogeneous conditions, the homogeneous-heterogeneous approach may underestimate the effects of physiological integration (Wang et al., 2009). In other words, both approaches compare and analyze the effects from only the perspective of the presence or absence of physiological integration. Due to limited growth space, growth time, and the microenvironment, no reproductive ramets are observed in most greenhouse experiments. Under homogeneous conditions, we first confirmed the important role of physiological integration in plasticity in sexual reproduction in clonal plants through *in situ* field experiments. Our approach is simple and convenient and can be widely used to study the effects of physiological integration on plasticity in sexual reproduction in rhizomatous, stoloniferous, and cespitose clonal plants in the future.

### Nutrient Translocation During Reproductive Development

The translocation of resources to emerging ramets and the significant role of physiological integration in the early development of new ramets (e.g., Hartnett and Bazzaz, 1983; Alpert, 1996; Xu et al., 2012), as well as marked changes in patterns of resource translocation from maternal ramets to daughter ramets across ramet ontogeny, are well documented in the literature (Duchoslavová and Jansa, 2018), but studies on the translocation of resources between vegetative ramets and reproductive ramets are lacking. Although only one study found that vegetative ramets could transfer their nutrients to adjacent reproductive ramets through the rhizome at the flowering stage in an ornamental clonal plant *Iris laevigata* (Wang et al., 2017b), the research focused on a single point in reproductive development. It is unclear how translocation of nutrients changes with the process of reproductive development. In the present study, we found significant differences in the amount of translocated ^15^N in the leaf blade, sheath, stem, inflorescence, and whole ramet of the reproductive ramet in *L. chinensis* among the heading, flowering, milk-ripe, and full-ripe stages (**Tables 3** and **4**). Among the reproductive ramets with different connection forms of vegetative ramets, the amount of translocated ^15^N in the leaf blade, sheath, stem, inflorescence, and whole ramet first increased and then decreased, and the amount in all parts was largest at the milk-ripe stage and smallest at the heading or full-ripe stage (**Figures 2** and **3**), which supported our second hypothesis. Under natural conditions, the reproductive ramets of *L. chinensis* enter the reproductive growth stage after heading at the end of May. According to source-sink theory, once the plant begins reproductive growth, continuous apical dominance appears (Cline, 1997; Wuest et al., 2016). At the level of organs, leaves perform photosynthesis and are the “source” due to nutrient production, while inflorescences produce seeds and are the “sink” due to nutrient accumulation. At the level of clonal fragments (groups of connected ramets), a source-sink relationship in which the reproductive ramet is the center is formed between vegetative and reproductive ramets. The vegetative ramet is the “source” due to nutrient production, while the reproductive ramet is the “sink” due to nutrient accumulation. With the progression of sexual reproduction, the strength of this source-sink relationship reached a peak at the milk-ripe stage, when the nutrient accumulation was most vigorous, in *L. chinensis*.

### Direction and Position Order Influence Nutrient Translocation

In clonal plants, there are two major modes of resource translocation: acropetal translocation (from developmentally older to younger ramets) and basipetal translocation (from developmentally younger to older ramets) (Alpert and Mooney, 1986; Alpert, 1996). Most studies have shown that acropetal translocation is more common than basipetal translocation within clonal plants (Alpert, 1996; de Kroon et al., 1996; D’hertefeldt and Jónsdóttir, 1999). Studies on rhizomatous *Carex bigelowii* and stoloniferous *Fragaria chiloensis* found that nitrogen was translocated predominantly to the younger ramets, but basipetal translocation was also detected within a clone (Jónsdóttir and Callaghan, 1990; Alpert, 1996). Previous experiments concerning resource translocation in *L. chinensis* used only a simple system of two interconnected vegetative ramets (Gao et al., 2014; Zhou et al., 2014). However, under natural conditions, there are usually more complex connection systems in *L. chinensis* populations, such as clonal systems with three members (two reproductive ramets and one spacer vegetative ramet connected by one and the same rhizome) or five members (one reproductive ramet and four vegetative ramets connected *via* one and the same rhizome). In the present study, we detected significant effects of transfer direction on the amount of translocated ^15^N in the leaf blade, sheath, stem, inflorescence, and whole ramet of the reproductive ramet in *L. chinensis* with a three-member clonal system (**Table 3**). At each reproductive stage, when the spacer ramet was labeled with ^15^N, the amount of translocated ^15^N in the acropetal reproductive ramet was larger than that in the basipetal ramet. The amount of translocated ^15^N in the leaf blade, sheath, stem, inflorescence, and whole ramet of the acropetal reproductive ramet was larger than that in the corresponding parts of the basipetal ramet at the milk-ripe stage (**Figure 2**). These results supported our third hypothesis. According to source-sink theory, after two reproductive ramets in a three-member clonal system begin simultaneous reproductive growth, the spacer vegetative ramet acts as the “source” to simultaneously supply nutrients to the two reproductive ramets. The sink strength of the two reproductive ramets determines the amount of translocated nutrients. We found that the acropetal reproductive ramet obtained more nutrients, indicating that the acropetal ramet was the stronger recipient. The translocation of a large amount of nutrients to the apex of the rhizomes helps clonal plants expand a wider space.

For the five-member-clonal system of one basipetal reproductive ramet and four vegetative ramets connected *via* one and the same rhizome, significant effects of the position order of the connected vegetative ramet on the amount of translocated ^15^N were found in the leaf blade, sheath, stem, inflorescence, and whole ramet of basipetal reproductive ramets of *L. chinensis* (**Table 4**). At each reproductive stage, with increasing position order of the connected acropetal vegetative ramet, the amount of translocated ^15^N in the leaf blade, sheath, stem, inflorescence, and whole ramet of the basipetal reproductive ramet exhibited a significant linear, exponential or logarithmic decrease (**Figure 4**), which supported our fourth hypothesis. There are two possible reasons for this decrease in nutrient supply. On the one hand, nutrient allocation is limited by transport distance (Pan, 2012). With an increase in the position order of the connected vegetative ramet, the distance between the vegetative ramet and the reproductive ramet increased, and the amount of ^15^N translocated to the reproductive ramet consequently decreased. A negative curvilinear relationship was observed between the amount of ^15^N in the apical tillers and the distance to roots supplied with the isotope in *C. bigelowii* (Jónsdóttir and Callaghan, 1990), reflecting the importance of proximity in nutrient translocation. On the other hand, nutrient allocation is limited by source strength (Pan, 2012). Duchoslavová and Jansa (2018) found that the amount of ^15^N translocated from daughters to mothers increased during ramet development in the stoloniferous clonal plant *Agrostis stolonifera*. By this token, with an increase in the position order, the acropetal vegetative ramets became increasingly young in terms of development. The younger vegetative ramets were at the vigorous growth stage and thus in need of nutrients; thus, they transferred small amounts of nutrients to the basipetal reproductive ramet.

## Conclusions

In clonal populations of *L. chinensis* growing in a homogeneous environment, with an increase in the number of vegetative ramets connected to tillering nodes, ramet biomass, inflorescence biomass, seed number, the seed-setting percentage, reproductive allocation, and reallocation as well as their respective PPIs increased. The difference in nutrient supply caused by the number of connected vegetative ramets directly affected phenotypic plasticity in sexual reproduction. The nutrients supplied from vegetative ramets to reproductive ramets were affected by the reproductive stage, transfer direction and position order of the vegetative ramets. Different connection forms of vegetative ramets translocated the largest amount of nutrients to the reproductive ramet at the vigorous milk-ripe stage, they translocated a larger amount of nutrients to the acropetal reproductive ramet than to the basipetal reproductive ramet, and the closer to the reproductive ramet they were, the larger the amount of nutrients they translocated was.

## Data Availability Statement

The datasets generated during and/or analyzed during the current study are available from the corresponding author on reasonable request.

## Author Contributions

YY and HL designed the experiments. JG performed the experiments. JG and HL analyzed the data. JG, HL and YY wrote the manuscript. All authors read and approved the manuscript.

## Funding

This research was supported by the Natural Key Research and Development Program of China (2016YFC0500602), National Natural Science Foundation of China (31672471 and 31670427), and the Program for Introducing Talents to Universities (B16011).

## Conflict of Interest

The authors declare that the research was conducted in the absence of any commercial or financial relationships that could be construed as a potential conflict of interest.
